# Ectomycorrhizal Community Structure of *Salix* and *Betula* spp. at a Saline Site in Central Poland in Relation to the Seasons and Soil Parameters

**DOI:** 10.1007/s11270-015-2308-7

**Published:** 2015-03-19

**Authors:** Katarzyna Hrynkiewicz, Sonia Szymańska, Agnieszka Piernik, Dominika Thiem

**Affiliations:** 1 Department of Microbiology, Faculty of Biology and Environmental Protection, Nicolaus Copernicus University, Lwowska 1, 89-100 Torun, Poland; 2 Faculty of Biology and Environmental Protection, Nicolaus Copernicus University, Lwowska 1, Toruń, Poland

**Keywords:** Salinity, Diversity, Ectomycorrhizal fungi, Willow, Birch

## Abstract

Saline stress is one of the most important abiotic factors limiting the growth and development of plants and associated microorganisms. While the impact of salinity on associations of arbuscular fungi is relatively well understood, knowledge of the ectomycorrhizal (EM) fungi of trees growing on saline land is limited. The main objective of this study was to determine the density and diversity of EM fungi associated with three tree species, *Salix alba*, *Salix caprea* and *Betula pendula*, growing in saline soil during two seasons, autumn and spring. The site was located in central Poland, and the increased salinity of the soil was of anthropogenic origin from soda production. The degree of EM colonisation of fine root tips varied between 9 and 34 % and depended on the tree species of interest (*S. caprea* < *S. alba* < *B. pendula*) and season (spring < autumn). Moreover, the ectomycorrhizal colonisation of *B. pendula* was positively correlated with pH and CaCO_3_, while for *S. caprea* and *S. alba,* colonisation was associated with most of the other soil parameters investigated; e.g. salinity, C_org_ and N. Analysis of EM fungi revealed four to five different morphotypes per each season: *Tomentella* sp. Sa-A, *Hebeloma collariatum* Sc-A, *Geopora* sp. Sc-A, *Helotiales* sp. Bp-A in the autumn and *Tomentella* sp. Sa-S, *Tomentella* sp. Sc-S and three morphotypes from the families Thelephoraceae and Pyronemataceae in the spring. In conclusion, the density of EM is related to the level of salinity (EC_e_), season and tree species. *Tomentella* spp., *Hebeloma* sp., *Geopora* sp. and *Helotiales* sp. are groups of species highly adapted to saline conditions.

## Introduction

Symbiosis of ectomycorrhizal (EM) fungi is a commonly known type of plant association responsible for the proper growth and development of trees under abiotic stress conditions (Hrynkiewicz and Baum 2012a). Well-adapted ectomycorrhizae can increase the availability of nutrients and water to the host plant in adverse soil conditions (e.g. poor and drought-affected soils), decrease the direct negative impact of soil contamination (e.g. heavy metals such as Zn, Cu, Mn, Ni and Cr) and unfavourable pH and increase the resistance to disease from pathogenic fungi and bacteria (Dell 2002; Paradi and Baar 2006). The density and diversity of certain types of EM fungi in abiotic stress conditions can significantly depend on the tree species, seasonal changes and type of biotic and/or abiotic stress (Hrynkiewicz et al. 2008, 2010a, b). High soil salinity is an abiotic factor that may have a negative impact on EM associations, and research into understanding its influence is on-going.

Soil salinity is a serious problem for plant growth and development and may lead to a reduction in the abundance and metabolic activity of microorganisms (Silva Maganhotto de Souza and Fay 2012; Milosević et al. 2012; Szymańska et al. 2013). According to the Food and Agriculture Organization (FAO 2008), saline soils represent more than 6 % of the world's land and is one of the fastest processes causing land degradation (Kõljalg et al. 2000). This is the consequence of the destruction of the soil structure and therefore a reduction in oxygen, as a result of the accumulation of water-soluble salts in the soil (mainly the accumulation of Na^+^ but also K^+^, Mg^2+^, Ca^2+^, Cl^−^, SO_4_^2−^, CO_3_^2−^ and HCO_3_^−^ ions) (Silva Maganhotto de Souza and Fay 2012). Previous studies focused primarily on halotolerant plants and associated halophilic/halotolerant bacteria and endomycorrhizal fungi (Evelin et al. 2009; Gago et al. 2011; Silva and Fay 2012; Milosević et al. 2012; Szymańska et al. 2013). It is already known that arbuscular mycorrhizal (AM) fungi can promote the growth of crops in saline agricultural soils (e.g. effect of fertilisation) (e.g. Aggarwal et al. 2012). Meanwhile, much less attention has been paid to associations of EM fungi and plants growing at saline sites (Dell 2002; Bois et al. 2006; Jimenez-Casas and Zwiazek 2013).

*Salix* and *Betula* spp. are pioneer tree species that naturally grow in harsh soil conditions (Varga et al. 2009; Hrynkiewicz et al. 2009a, 2010a, b) and are the only tree species naturally growing in the saline area analysed in this study. The extraordinary properties of these trees to unfavourable soil conditions could be the result of the ease and simplicity in the development of EM symbiosis on the roots of these species. We hypothesised that EM fungi, characterised by a high tolerance to unfavourable abiotic conditions, can be a key factor in the protection of the host plants growing in saline soils (i) and that EM community structure may depend on the season and correlate with salt level and other soil parameters (ii).

The aim of our research was to determine the density and diversity of EM fungi associated with different tree species (*S. alba*, *S. caprea* and *B. pendula*) growing in saline soil in relation to seasonality and changes in salt concentration and other soil parameters.

## Materials and Methods

### Site Description and Sampling

This study was carried out in autumn 2012 and spring 2013 in Inowrocław-Mątwy (N 52° 48, E 18° 15) in the central part of Poland. This region has a continental climate with a mean annual temperature of +8.3 °C and a mean annual precipitation of 494 mm. The area of our research (ca 100 ha) included a saline meadow located near a soda factory (Soda Poland CIECH SA). It is the only company in Poland and the second company in the European market that produces dense and light soda ash. The factory has been in operation since 1879. Local land degradation was a result of the improper storage of industrial wastes from soda production (Hulisz and Piernik 2013). The only trees naturally growing in this area are *Salix alba*, *Salix caprea* and *Betula pendula* (~20 years old, growing at a distance 20–30 m). The soils degraded by the technogenically induced salinisation process in Inowrocław-Matwy are classified as Mollic Technosoils (Calcaric) (Hulisz and Piernik 2013).

Root and soil samples (20 × 20 cm, 20 cm deep) of the trees were collected in two seasons: autumn 2012 (A) and spring 2013 (S). In each season, nine root and soil samples were collected from three plants. In total, 18 root and soil samples were taken (two seasons, three plant species, each variant in three replications).

Due to the fact that in the study area, naturally occurred only in three trees (as described above), we were not able to collect a larger number of natural samples/reps. However, in view of the pioneering nature of research on abundance and diversity of ectomycorrhizae at saline areas and the relatively high sensitivity of trees that naturally colonise the saline lands, our findings may help to initiate further research, which confirm the possibilities of creating mycorrhizal association under high salinity.

### Soil Description

Rhizosphere soil (soil closely adjacent to the plant roots) of each sample was gently separated from the roots and analysed. Conventional physico-chemical analysis of the soil rhizosphere was conducted to determine the impact of soil on the abundance and diversity of EM fungi. Within the basic analysis, the concentrations of organic matter, organic carbon and calcium carbonate were determined according to methods described by Bednarek (2005). The total nitrogen, phosphorus soluble in 1 % citric acid solution (P_ca_), pH-H_2_O and pH-KCl levels, the salinity of a saturated paste (expressed as electrical conductivity (EC_e_)) and the concentration of major anions (Cl^−^, SO_4_^2−^, HCO_3_^−^) and cations (K^+^, Ca^2+^, Na^+^, Fe^2+^) were determined based on methods described by van Reeuvijk (2002) (Table 3).

### Determination of Density and Diversity of EM Morphotypes

Analysis was performed according to Hrynkiewicz et al. (2012b). Shortly, root samples with soil were soaked in de-ionised water overnight. Then, the roots were gently separated and washed using de-ionised water before microscopic examination (Carl Zeiss, Jena, Germany). From each of the 18 soil samples, from six to ten root fragments (10 × 10 cm for each sample from autumn and *S. caprea* roots from spring; 6 × 10 cm roots of *S. alba* and 7 × 10 cm roots of *B. pendula* from spring) were randomly chosen on a grid for microscopic quantification of EM colonisation of fine root tips. The number of living non-colonised root tips vs. visually colonised EM root tips was counted using the formula: root tips × 100 %/total numbers of root tips (Agerer 1991). In total, 17,513 root tips were scanned. A minimum of 443 to 819 root tips per sample and 1035 to 5309 roots per tree species and season were investigated. All colonised root tips collected from each sample were used separately for analysis of EM fungal species diversity. In total, 914 EM root tips were collected. A minimum of 11 to 16 EM root tips per sample and of 99 to 213 EM root tips per tree species (*S. caprea*, *S. alba*, *B. pendula*) and season (autumn 2012 and spring 2013) were investigated. The morpho-anatomical EM fungal types were distinguished by macroscopic characteristics of the fungal mantle, colour, surface appearance, presence of emanating hyphae and hyphal strands, as well as microscopic features such as mantle type and hyphal connections (Agerer 1987–2002). In total, nine different EM morphotypes were identified from all samples. Two to five root tips per morphotype found in each analysed sample were separately frozen in Eppendorf tubes and stored at −20 °C for molecular analysis.

## Molecular Analysis

DNA was extracted from the EM root tips using the plant DNAeasy Plant Mini Kit (Qiagen) according to the protocol. The fungal taxa were identified based on the internal transcribed spacer (ITS) region of the rDNA. To amplify this region, the fungal specific primers ITS1F and ITS4 were used (Gardes and Bruns 1993). The PCR analysis and the DNA sequencing were conducted according to Hrynkiewicz et al. (2009a, b). The forward and reverse sequences were assembled and edited using Sequencher 5.1 (Gene Codes 20). The identification process required a minimum of 98 % similarity to the investigated sequence, with reference sequences deposited in GenBank and/or the UNITE nucleotide database. All DNA sequences determined in this research will be submitted to GenBank, and the accession numbers will be presented in Table 4.

### Statistical Analysis

Differences in the abundance of EM fungi for all research variants (including different tree species and seasons) were investigated by ANOVA with Tukey's test as a post hoc comparison using Statistica software (Statistica ver. 7, Statsoft). The relation between the level of root colonisation of three tree species (*S. alba, S. caprea, B. pendula*) and soil properties during two seasons (autumn 2012 and spring 2013) was analysed by redundancy analysis (RDA). The same analysis was used to assess the relation between root colonisation by EM fungi and rhizosphere soil properties. The relation between the most important variable of chemical rhizosphere soil parameters of the tree species at the saline site during two seasons was analysed by discriminant analysis (canonical variate analysis (CVA)). The relative importance and statistical significance of each environmental factor in the ordination model were tested by a forward selection procedure and Monte Carlo permutation test. All ordination methods were applied with the use of the Canoco 4.5 package (ter Braak and Smilauer 2002).

## Results

### Rhizosphere Soil Parameters

The physico-chemical parameters of soil closely adjacent to the roots differed significantly between the tree species (*B. pendula*, *S. alba*, *S. caprea*) and between the seasons (autumn 2012 and spring 2013) (Table 1). In general, the highest average percentages of organic matter (OM), organic carbon (C_org_) and total nitrogen (N_tot_) were observed in the rhizosphere soil of *S. alba*, while significantly lower values were noted in the rhizospheres of *B. pendula* and *S. caprea*. Otherwise, the highest levels of C/N, pH and CaCO_3_ were observed in the rhizosphere soil of *B. pendula*, with significantly lower values observed in the case of *S. caprea* and *S. alba* (Table 1). Significant differences in soil parameters between the two seasons were tree-specific (significant increase marked with an arrow).

**Table 1:** Physico-chemical soil parameters (mean and standard deviation) in autumn 2012 and in spring 2013

Variable	Tree species	Autumn 2012	Spring 2013
Org. matter (MO) (g kg^−1^)	*Salix alba*	20.51 c (0.79)	26.62 c [↑] (1.29)
	*Salix caprea*	9.53 b [↑] (0.60)	7.01 a (0.59)
	*Betula pendula*	9.33 (0.61) a	10.2867 (0.75) b
C org. (g kg^−1^)	*S. alba*	20.51 b [↑] (0.95)	10.67 b (1.58)
	*S. caprea*	9.53 a [↑] (0.03)	4.44 a (0.13)
	*B. pendula*	9.33 a [↑] (0.10)	3.81 a (0.20)
N total (g kg^−1^)	*S. alba*	0.73 a (0.04)	0.90 c [↑] (0.04)
	*S. caprea*	0.41 a [↑] (0.01)	0.20 a (0.00)
	*B. pendula*	0.20 a (0.0099)	0.26 b [↑] (0.0074)
C/N	*S. alba*	14.64 b (0.45)	17.76 a [↑] (0.99)
	*S. caprea*	10.87 a (0.24)	17.61 a [↑] (0.86)
	*B. pendula*	18.78 c (1.27)	17.91 a (0.25)
pH-H_2_O	*S. alba*	7.73 a [↑] (0.11)	7.45 a (0.05)
	*S. caprea*	8.10 b (0.00)	8.07 b (0.06)
	*B. pendula*	8.50 c [↑] (0.00)	7.87 c (0.06)
pH-1 M KCl	*S. alba*	7.57 a (0.15)	7.35 b (0.05)
	*S. caprea*	7.80 b (0.00)	7.73 a (0.06)
	*B. pendula*	8.00 c (0.00)	7.70 a (0.00)
CaCO_3_ (g kg^−1^)	*S. alba*	5.07 a (0.11)	10.65 b [↑] (0.15)
	*S. caprea*	19.57 b [↑] (0.76)	10.50 a (0.20)
	*B. pendula*	46.03 c (1.62)	43.23 c (2.16)
Na^+^ (mg l^−1^)	*S. alba*	101.8 (2.7)	433.7 (11.5)
	*S. caprea*	33.0 (0.9)	257.7 (6.8)
	*B. pendula*	89.7 (2.4)	280.2 (7.4)
Ca^2+^ (mg l^−1^)	*S. alba*	287.5 (7.6)	385.1 (11.6)
	*S. caprea*	60.8 (2.7)	197.0 (8.6)
	*B. pendula*	168.2 (7.0)	89.9 (3.5)
K^+^ (mg l^−1^)	*S. alba*	205.7 (7.4)	174.3 (6.1)
	*S. caprea*	33.1 (1.3)	53.2 (1.9)
	*B. pendula*	48.1 (2.1)	33.9 (1.0)
Mg^2+^ (mg l^−1^)	*S. alba*	34.9 (0.7)	62.4 (1.9)
	*S. caprea*	8.77 (0.3)	23.8 (1.0)
	*B. pendula*	15.9 (0.7)	14.0 (0.5)
Fe^2+^ (mg l^−1^)	*S. alba*	1.22 (0.0)	0.87 (0.0)
	*S. caprea*	0.98 (0.0)	0.99 (0.0)
	*B. pendula*	1.25 (0.0)	1.13 (0.0)
Cl^−^ (mg l^−1^)	*S. alba*	716.5 (29.3)	1600 (80.2)
	*S. caprea*	160.0 (4.4)	628.0 (31.3)
	*B. pendula*	401.0 (7.8)	590.0 (12.1)
SO_4_^2−^ (mg l^−1^)	*S. alba*	283.9 (6.9)	69.8 (3.4)
	*S. caprea*	53.4 (1.7)	290.1 (9.6)
	*B. pendula*	146.4 (6.6)	109.6 (2.4)
HCO_3_^−^ (mg l^−1^)	*S. alba*	16.3 (1.0)	11.4 (0.3)
	*S. caprea*	8.1 (0.2)	9.8 (0.6)
	*B. pendula*	12.2 (0.3)	8.9 (0.3)

The content of the components in a saturated extract. The data represent the mean of nine replicates ± SD. The mean values of each parameter within the given column marked with the same letter do not differ significantly (*p* < 0.05)

“[↑]” significantly higher level of rhizosphere soil parameter observed between the seasons

**Fig. 1:**
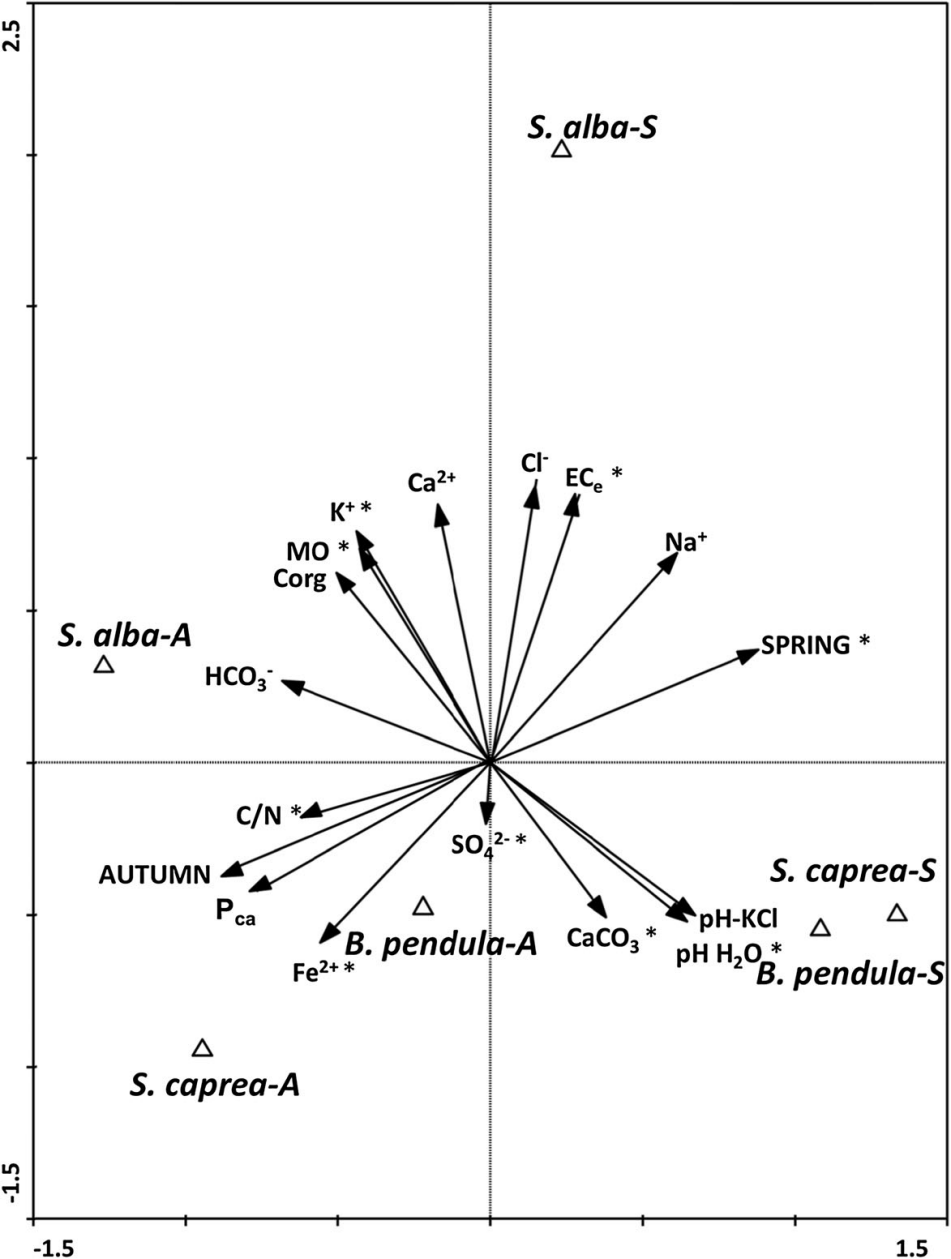
Canonical variate analysis: diagrams with axes 1 and 2 for 15 chemical soil parameters (MO, C org, C/N, pH-H_2_O, pH-1 M KCl, CaCO_3_, EC_e_, Ca^2+^, K^+^, Na^+^, Fe^2+^, HCO_3_^−^, Cl^−^, SO_4_^2−^, P_ca_) of three tree species (*S. alba, S. caprea, B. pendula*) at a saline site during two seasons (autumn 2012 and spring 2013). *p ≤ 0.05, significant factors

The level of salinity (EC_e_) in the rhizosphere soils ranged from 0.5 to 5.0 (dS m^−1^) and was the highest in the rhizosphere of *S. alba* (2.8 and 5.0 dS m^−1^ for autumn and spring, respectively) and the lowest in the rhizosphere of *B. pendula* (0.5 and 2.0 dS m^−1^ for autumn and spring, respectively). The salinity of the rhizosphere of *S. caprea* was at the average level (1.4 and 2.4 dS m^−1^ for autumn and spring, respectively). According to soil scale salinity (Jackson 1958), the rhizosphere samples belong to slightly saline soils (with the exception of *B. pendula* in autumn, when the salinity was <2 dS m^−1^ - non-saline soil). Analysis of soil nutrient concentrations revealed the highest levels of the parameters Ca^2+^, Mg^2+^, K^+^, Na^+^, Cl^-^, HCO^3−^ and SO_4_^2−^ (mg l^−1^) for *S. alba*; Fe^2+^ (mg l^−1^) and P_ca_ (mg kg^−1^) for *S. caprea;* and the lowest in the rhizosphere soil of *B. pendula*. The level of soil nutrient concentrations (similar to the level of salinity) was in general higher during the spring than the autumn. The CCA diagram based on chemical soil properties in the rhizosphere soil (18 parameters in total) under the three tree species (*S. alba, S. caprea, B. pendula*) at a saline site during two seasons (autumn and spring) (Fig. 1) allowed the selection of nine factors (MO, C/N, CaCO_3,_ EC_e_, K^+^, Fe^2+^, P_ca_, spring) that are significant in the differentiation of soil properties. CCA analysis showed that axis 1 significantly contributed to the explained variance (*p* < 0.05), including the seasons. The soil parameters analysed in the autumn samples were positively related to the C/N, K^+^, Fe^2+^ and P_ca_ content of the soil, while the spring soil properties were associated with EC_e_, Na^2+^, pH and CaCO_3_ levels.

### Effect of Tree Species, Seasons and Soil Parameters on EM Colonisation in Saline Soil

**Fig. 2:**
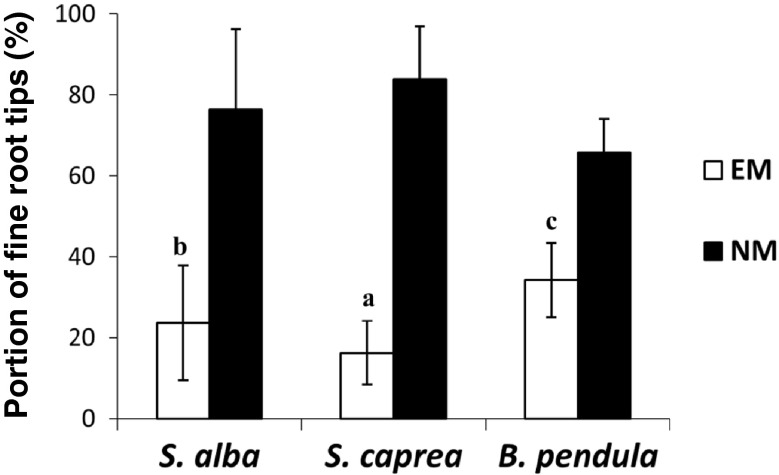
Ectomycorrhizal (*EM*) and non-mycorrhizal (*NM*) fine root tips (%, mean ± standard deviation) under *S. alba*, *S. caprea* and *B. pendula* in autumn 2012

**Fig. 3:**
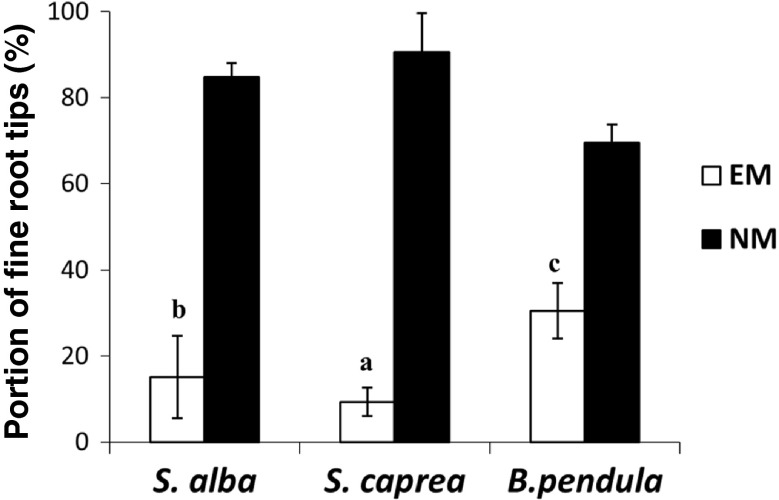
Ectomycorrhizal (*EM*) and non-mycorrhizal (*NM*) fine root tips (%, mean ± standard deviation) under *S. alba*, *S. caprea* and *B. pendula* in spring 2013

The EM colonisation of fine root tips in all treatments ranged from 16 to 34 % in the autumn and from 9 to 30 % in the spring (Figs. 2 and 3). In general, the highest portions of the EM roots were observed for *B. pendula* and the lowest for *S. caprea* (*B. pendula* > *S. alba* > *S. caprea*). The frequency of EM fine root tips in the total number of investigated fine root tips ranged from 30 to 34 % for *B. pendula*, 15 to 23 % for *S. alba* and 9 to 16 % for *S. caprea* in the spring and autumn, respectively (Figs. 2 and 3). The results of two-factorial ANOVA analysis and Tukey's test confirmed a significant effect of all variants analysed during the experiment: season, tree species and season × tree species on EM colonisation of fine root tips in saline soil (Tables 2, 3 and 4). The frequency of non-mycorrhizal fine root tips was always larger in the spring than in the autumn.

**Table 2:** Molecularly identified EM fungi on *Salix alba*, *Salix caprea*, and *Betula pendula* fine roots during autumn 2012 and spring 2013

Tree species and season	T bp	Closest BLAST match (accession numbers—NCBI* and/or UNITE**)	% similarity	Classified as	EM density
Autumn 2012					
*S. alba*	637	*Tomentella* isolate [EU668202 and UDB002428]* (Bidartondo and Read 2008); *Tomentella stuposa* [UDB011637]**	617/630 (97 %) and 587/595 (98 %) 623/639 (97 %)	*Tomentella* sp. Sa-A [KP745605]	24.63 %
*S. caprea*	699	*Hebeloma collariatum* [JQ724066]*	693/696 (99 %)	*H. collariatum* Sc-A [KP745607]	3.88 %
		*H. collariatum* [UDB015489]**	668/674 (99 %)		
	636	*Geopora* sp. [JQ724044]* (Hrynkiewicz et al. 2012b)	565/636 (89 %)	*Geopora* sp. Sc-A [KP745606]	11.82 %
		*Geopora arenicola* [UDB017620]**	368/406 (90 %)		
*B. pendula*	563	Uncultured fungus genomic [FN397282]* (Napoli et al. 2010)	563/566 (99 %)	*Helotiales* sp. Bp- [KP745604]	27.18 %
		Ectomycorrhizal fungus [JX043062]* (Karst et al. 2013)	542/552 (98 %)		
		*Helotiales* sp. [JN859267]* (Knapp et al. 2012)	528/541 (98 %)		
Spring 2013					
*S. alba*	874	Thelephoraceae [EF218829]* (Twieg et al. 2007)	831/874 (95 %)	*Tomentella* sp. Sa-S [KP745608]	10.82 %
		Thelephoraceae [AJ893343]* (Tedersoo et al. 2006b)	800/831 (96 %)		
		*Tomentella* sp. [UDB018687]**	832/866 (96 %)		
		*T. ellisii* [UDB016490]**	818/855 (95 %)		
*S. caprea*	623	*Tomentella ellisii* [DQ068971]* (Menkis et al. 2005)	598/614 (97 %)	*Tomentella* sp. Sc-S [KP745609]	7.54 %
		*Tomentella* sp. [UDB018687]**	583/614 (94 %)		
		*T. ellisii* [UDB016490]**	569/603 (94 %)		
*B. pendula*	–	Colour, brownish; rhizomorphs, not observed; mantle, plectemchymatic B; cystidia, lacking; surface, smooth; emanating hyphae, scarce	–	Thelephoraceae B.p_1S	6.95 %
	–	Colour, brown; rhizomorphs, not observed; mantle, plectemchymatic B; cystidia, lacking; surface, smooth; and emanating hyphae, lacking	–	Pyronemataceae B.p_2S	5.20 %
	–	Colour, gold-brown; rhizomorph,: not observed;, *mantle*, plectemchymatic A; cystidia, lacking; surface: smooth; emanating hyphae: abundant with clamps	–	Thelephoraceae B.p_3S	1.97 %

Abbreviations: *B.p Betula pendula, S.c Salix caprea, S.a Salix alba*

**Table 3:** Results of two-factorial ANOVA: MS effect, *F* value and *P* level for density of EM fungi observed for the two seasons (autumn 2012, spring 2013) and three tree species (*S. alba, S. caprea, B. pendula*)

Parameter	MS effect	*F*	*p* level
(1) Season	4418.1	47.01	0.000*
(2) Tree	15,606.5	166.04	0.000*
(3) Season × tree	404.2	4.30	0.014*
Error	94.0

**Table 4:** Tukey's test for testing all pairwise comparisons (season and tree species)

Tukey post hoc comparison	
(1) Season	
Autumn 2012	23.621 b
Spring 2013	16.053 a
(2) Tree	
*S. alba*	19.388 b
*S. caprea*	12.003 a
*B. pendula*	31.347 c

Significant differences are marked by different letters

**Fig. 4:**
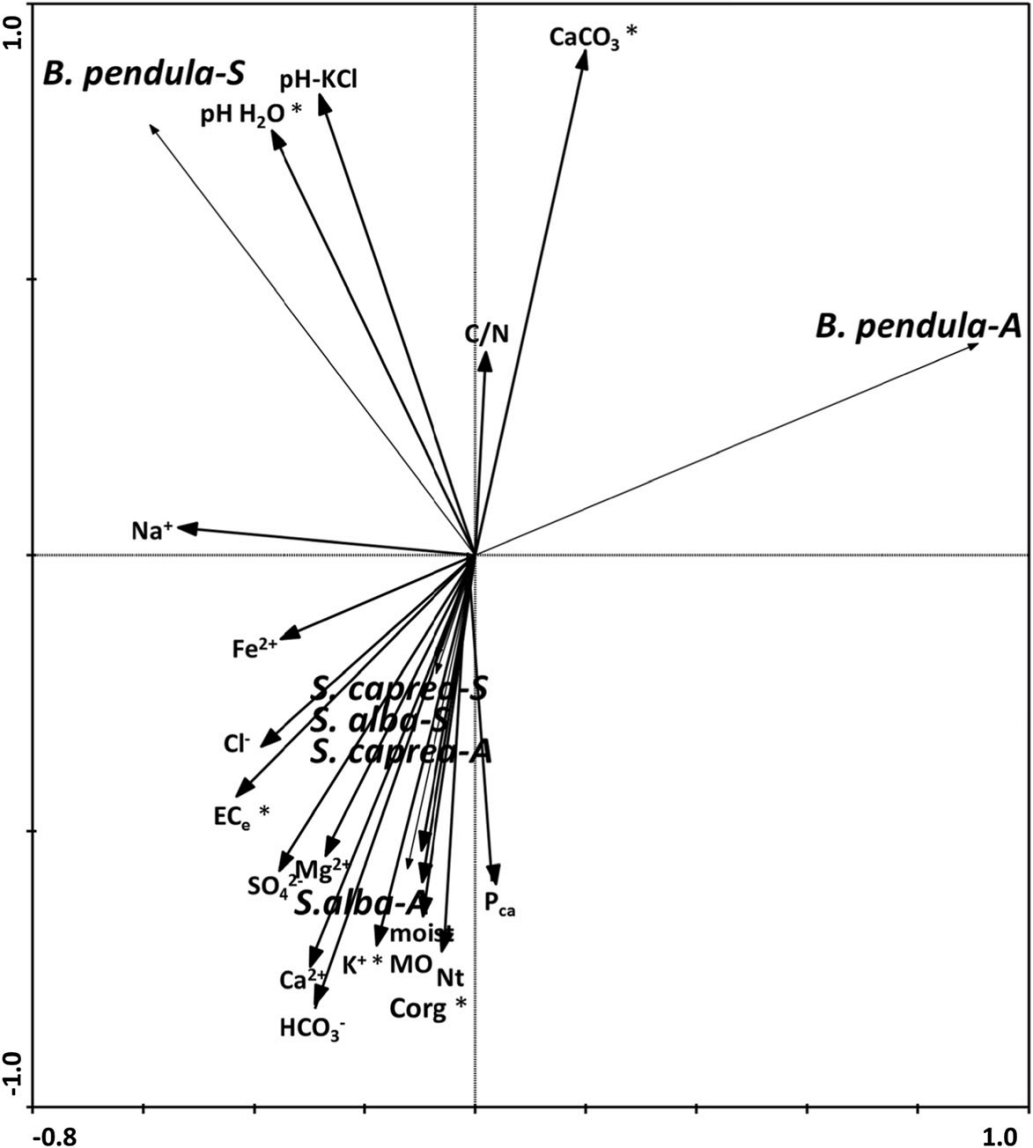
Redundancy analysis, diagrams with axes 1 and 2 for level of EM colonisation of fine roots of three tree species (*S. alba, S. caprea, B. pendula*) during two seasons (autumn and spring) and soil properties in the rhizosphere. *p ≤ 0.05, significant factors

The RDA diagram based on the level of colonisation of EM fungi of the tree species, seasons and chemical soil properties in the rhizosphere is shown in Fig. 4. The analysis allowed the selection of five factors (from 18 analysed) significant in differentiation of the density of EM fungi. The CaCO_3_ and related pH-H_2_O level ratio significantly contributed to explaining the density of EM fungi colonising *B. pendula* (both seasons), while the organic carbon, EC_e_ and K^+^ significantly contributed to explaining the amount of EM fungi associated with *S. alba* and *S. caprea* (both seasons) (Fig. 4).

### Diversity of EM Fungi in Relation to Different Tree Species, Seasons and Soil Parameters

Nine EM morphotypes were found in the investigated treatments, two on *S. alba* (one in each season), three on *S. caprea* (two in autumn and one in spring) and four on *B. pendula* (one in autumn and three in spring). One EM fungus was identified to the species level (*Hebeloma collariatum* Sc-A), five to the genus level (*Tomentella* sp. Sa-A, Sa-S, Sc-S, *Geopora* sp. Sc-A, *Helotialaes* Bp-A) and three morphotypes, which were identified on the basis of morpho-anatomical properties (Bp_1S, Bp_2S, Bp_3S). The number of different EM morphotypes per each variant of the experiment (three tree species and two seasons, six variants in total) ranged from one to three. The number of different morphotypes was higher in the spring than in the autumn (five and four, respectively). EM formation by Thelephoraceae (*Tomentella* sp. Sa-A, Sa-S and Sc-S) was observed for *S. alba* (both seasons) and *S. caprea* (spring) and by Pyronemataceae (*Geopora* sp. Sc-A, *Helotiales* sp. Bp-A) for *S. caprea* and *B. pendula* (in both cases only in the autumn). A representative of Cortinariaceae (*H. collariatum* Sc-A) was observed only on the roots of *S. caprea* (in autumn). Three morphotypes that were not identified molecularly were classified on the basis of their morphological and anatomical properties (Bp_1S, Bp_2S; Bp_3S, see Table 2).

**Fig. 5:**
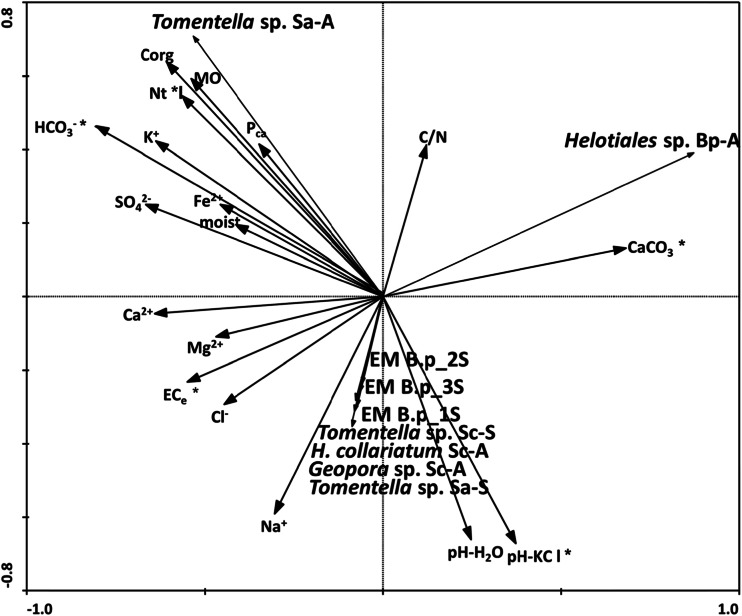
Redundancy analysis, diagrams with axes 1 and 2 for soil properties in the rhizosphere and level of EM colonisation of fine roots of three tree species (*S. alba, S. caprea, B. pendula*) during two seasons (autumn and spring). *p ≤ 0.05, significant factors

The RDA diagram based on 18 rhizosphere soil properties (similar to the case of RDA analysis made for EM density) and the frequency of EM morphotypes (nine in total) is shown in Fig. 5. Most of the EM types (*H. collariatum* Sc-A, *Geopora* sp. Sc-A, *Tomentella* sp. Sa-S, *Tomentella* sp. Sc-S, B.p_1S, B.p_2S, B.p_3S) were positively related with pH and EC_e_ level (significant effect). The other two EM morphotypes, associated with *S. alba* during the autumn (*Tomentella* sp. Sa-A) and with *B. pendula* during the autumn (*Helotiales* sp. Bp-A) were positively related to HCO_3_^−^, N_tot_ and CaCO_3_ levels in the soil, respectively (Fig. 5).

## Discussion

Well-adapted EM fungi can be fundamental for the growth and survival of the host plant in harsh soil conditions (Hrynkiewicz and Baum 2012a; Timling et al. 2012). The presence of EM fungi naturally associated with the roots of tree species (*S. alba, S. caprea, B. pendula*) growing in unfavourable saline soil supports our initial assumption and confirms their important role in the acclimatisation of hosts under saline stress. The level of EM colonisation (9–34 %) of fine root trees growing in saline conditions was similar to the EM colonisation observed at other disturbed test sites; e.g. *S. caprea* growing in a heavy metal-contaminated site: 3–36 % (Hrynkiewicz et al. 2008), mycorrhizal formation on roots from three willow clones (*Salix* spp.) on fly ash: approximately 3 %. The levels were, however, much lower compared with *S. repens* growing in unfavourable dune ecosystems: 62–87 % (van der Heijden et al. 1999), *S. caprea* at a heavy metal polluted site: 36–50 % (Regvar et al. 2010) or *Salix polaris* in the subarctic tundra: 35–64 % (Hrynkiewicz et al. 2009a). Ruotsalainen et al. (2009), who investigated EM colonisation along three environmental gradients (two natural and one with human-induced pollution) within the Kola Peninsula (NW Russia), revealed that total EM colonisation of mountain birch (*Betula pubescens* ssp. *czerepanovii*) ranged between 30 and 60 % and did not show stress-related patterns. Our results are in agreement with the observation of Ishida et al. (2009), who noted poor EM colonisation in Mongolian willow in alkaline-saline soil, where some of the root systems were not colonised by EM fungi at all, and more than 57.7 % of the fine root tips were not colonised by EM fungi. The relatively low EM colonisation of fine root tips level observed in this work corresponds to the observation that EM colonisation is problematic in some primary successional sites (Allen et al. 1992) and is regulated mostly by the magnitude of soil disturbance. For instance, very poor EM colonisation was observed on the roots of birch (*B. pubescens*) seedlings grown in soil from eroded sites in Iceland—an average number of EM roots less than 50 (Oddsdottir et al. 2010)—or in devastated volcanic deserts (Allen et al. 1992). Collecting the post-production waste products of the soda factory in ponds called “white seas” (beginning in 1882) led to a strong degradation of the test site analysed in this study. As a result, a strongly degraded area of approximately 135 ha is currently not used (Hulisz and Piernik 2013). The only herbaceous plants colonising areas with the highest levels of salinity are typical halophytes; e.g. *Salicornia europaea* and *Aster tripolium*. The trees analysed in this study are pioneer species that naturally colonise the periphery of the former settlers, suggesting that they have high tolerance to salt conditions. We suppose that the relatively low frequencies of ectomycorrhizae observed on the roots of *S. alba* and *S. caprea* are related mostly to the highest levels of salinity (EC_e_, 1.4–5.0 dS m^−1^) and concentrations of ions (e.g. Na^+^, Cl^−^, Ca^2+^, K^+^) in the rhizosphere of willow species compared with *B. pendula* (EC_e_, 0.53–1.97). Hyperosmotic stress in fungi is associated with inhibition of cell wall extension and cellular expansion, leading to a reduction in growth. Moreover, an excess of Na^+^ and/or Cl^−^ ions in fungal cells may alter enzymatic activity and protein and nucleic acid metabolism (Bois et al. 2006), thus decreasing the potential of EM fungi for successful colonisation of the plant roots. This statement may be supported by the observation that significantly lower EM colonisation of fine root tips occurred in the tree species during the spring, correlated with higher salt concentrations in this season. Soil salinity varies from season to season due to variations in hydrological parameters; e.g. the amount of rainwater and groundwater level. Ferjani et al. (2013) reported that the highest value of electrical conductivity (ECe) reached during the irrigation season (7.7 dS m^−1^) was decreased to 3.1 dS m^−1^ following the fall rains. In Polish climatic conditions, due to percolative type of the water regime, salt accumulation does not take occur, unlike arid and semi-arid climates. Therefore, the salinity level of the analysed soils was closely linked to the groundwater level (Hulisz and Piernik 2013). The greatest intensity of the salinisation process by capillary rise and evapotranspiration occurs when groundwater is present in a zone called the critical depth, where fluctuations of the groundwater level are relatively small (Hulisz and Piernik 2013). However, the variability of soil salinity at the microscale not only results from the saline water supply but also is favoured by the microrelief and some soil properties (organic matter content and texture—expressed as saturation percentage) (Hulisz and Piernik 2013). The high organic matter content allows for high ion accumulation and therefore higher salinity levels.

Many studies have shown that a high level of P in the soil has a negative effect on the formation of ectomycorrhizae (see reviews by Wallander 1995). In the rhizosphere of *S. alba* and *S. caprea*, with a significantly lower EM colonisation of fine root tips level, we have noted higher concentrations of P compared with *B. pendula*. Reductions in EM colonisation of fine root tips by *Salix viminalis* and *B. pendula* seedlings under elevated P levels was observed by Jones et al. (1991) and Newton and Pigott (1991), respectively. Puttsepp et al. (2004) reported that a higher abundance of EM fungi is associated with a lower pH, lower levels of K and P and higher levels of N and organic matter content. Because the highest density observed on the roots of *B. pendula* in our work was correlated with lower levels of K (33.1–33.9 mg/l) and P_ca_ (155–212 mg kg^−1^) compared with *S. alba* and *S. caprea*, but at the same time represented higher pH (7.87–8.50 in H_2_O, 7.70–8.00 in KCl), lower levels of N (0.2033–0.2617 %) and organic matter (9.3267–10.2867 %) compared with willow samples, we suggest that differences in the level of salt concentration have a large impact on EM density in saline soils.

Seasonal variability and accompanying changes in the weather can affect populations of EM fungi associated with the host plant (Walker et al. 2008). The necessity for studying systematic seasonal patterns of EM fungi is important because of impending global climate change. In our study, we observed a significantly higher abundance of EM fungi in the autumn (16–34 %) when the salinity of rhizosphere soil was definitely lower, compared with (9–30 %) the spring. The same tendency for willow (*S. viminalis*) and poplar (*Populus nigra × maximowiczii*) cultivated as short rotation coppice (SRC) was observed by Hrynkiewicz et al. (2010a): an average of 55 and 25.5 % in autumn and spring, respectively. However, *S. caprea* growing in heavy metal stress conditions revealed a higher density of EM fungi in the spring (23–36 %) than in the autumn (3–30 %) (Hrynkiewicz et al. 2008). The pattern of EM fungi frequency associated with *Alnus acuminata* (Betulaceae) occurring at two natural forests was not correlated with the seasons (EM colonisation of fine root tips range from 30.3 to 94 %) (Becerraa et al. 2005).

Our studies describe for the first time the density of EM fungi at saline areas, but there are many reports describing the role in alleviation of salt stress in plants by arbuscular mycorrhizal (AM) fungi (e.g. Evelin et al. 2009; Aggarwal et al. 2012). Salinity may directly and/or indirectly affect the development and functioning of AM fungi; e.g. by inhibition of spore germination and hyphal growth (Campagnac and Khasa 2014). Füzy et al. (2008) revealed that AM colonisation of salt aster (*A. tripolium*) and sea plantain (*Plantago maritima*) was greatest in late spring to early summer and had a second peak later in the autumn. Arbuscule formation and overall mycorrhizal colonisation appeared to be inversely correlated with the intensity of rainfall and suggest that, in addition to seasonality, drought may play an important role in governing AM activity in saline habitats. Furthermore, proper management of AM symbiosis has the potential to improve the profitability and sustainability of salt tolerance by host plants by minimising the movement of Na^+^ to the shoot and in general improving water and nutrient uptake (Aggarwal et al. 2012). It is quite probable that EM fungi possess the same properties, so that in higher salt stress conditions, these processes would intensify. Improved plant productivity of *Populus* spp. under salt stress conditions associated with proper EM fungi was emphasised by Luo et al. (2009). Comparative metabolite and transcriptome analysis in EM and non-EM roots of grey poplar revealed higher levels of myoinositole, abscisic and salicic acid, and K^+^ and Na^+^ in EM roots (Luo et al. 2009).

The diversity of EM fungi, similar to EM density, can be affected by numerous biotic (e.g. coexistence with bacteria, host genotype) and abiotic (e.g. heavy metal contamination, nutrient deficiency, pH) factors (Chai et al. 2013). Many authors emphasise the limiting effect of abiotic stress on the diversity of EM fungi compared with their total density (Paradi and Baar 2006; Hrynkiewicz et al. 2008). In this study, the *Salix* and *Betula* species growing in saline soil harboured nine different morphotypes of EM fungi. Four and five different morphotypes were observed during the autumn and spring, respectively, and the number of morphotypes associated with three different tree species (*S. alba, S. caprea, B. pendula*) depended on two seasons: three morphotypes on the roots of *B. pendula* during the spring, two morphotypes on *S. caprea* during the autumn and one EM morphotype per each other variant of the experiment. Those numbers of morphotypes are in line with the numbers reported for other *Salix* spp.; e.g. 11 EM fungal species associated with *Salix linearistipularis* growing in alkaline-saline soil (Ishida et al. 2009), 12 EM fungal partners colonising the root tips of *S. alba* growing in three riparian edge forests (Paradi and Baar 2006), 11 EM fungal partners identified from *Salix* clones in SRC in Estonia (Puttsepp et al. 2004), 14 EM fungal partners of *S. caprea* identified at heavy metal contaminated sites (Hrynkiewicz et al. 2008) and 7 fungal taxa identified from *S. viminalis* grown in SRC at an arable site in Germany (Hrynkiewicz et al. 2010a). Despite significant differences in the parameters of the soil, we have not observed a clear influence of seasonality on the diversity of EM fungi in our study. However, such observations have been made in other cases; e.g. oak seedlings (Walker et al. 2008). The EM fungal taxa were dominated by Basidomycetes (six of nine identified morphotypes): *Tomentella* sp. Sa-A, Sa-S and Sc-S, *H. collariatum* Sc-A, Thelephoraceae Bp-1S, Bp-2S with only three representatives (three of nine identified morphotypes) of Ascomycotes: *Geopora* sp. Sc-A and *Helotiales* sp. Bp-A and Pyrenomataceae Bp-3S. The dominance of EM fungal taxa from Basidiomycetes to Ascomycetes is in line with our earlier observations (Hrynkiewicz et al. 2008, 2009a) and the results of other authors (e.g. Timling et al. 2012). Fungal species from the family Thelephoraceae (*Tomentella* sp.) were observed on the roots of all tree species and in both seasons, while two representatives of Ascomycetes (*Geopora* sp. and *Helotiales* sp.) were characteristic mostly of autumn.

Thelephorales (*Tomentella* sp. Sa-A, Sa-S and Sc-S) were observed on the roots of all of the investigated tree species (on the roots of *B. pendula* identified on the basis of morpho-anatomical features—Thelephoraceae) and constituted the most numerous group of EM symbionts along with the highest level of EM colonisation of fine root tips (49.94 % in general, 100 % of Sa-A and Sa-S, 49.19 % of the EM fine roots for *B. pendula*). This agreed with the observed dominance of Thelephoraceae as EM fungal partners of *S. caprea* at heavy metal contaminated sites (Hrynkiewicz et al. 2008), *S. alba* in riparian edge forests (Paradi and Baar 2006) and *S. viminalis* grown in SRC at an arable site in Germany (Hrynkiewicz et al. 2010a). The characteristic colour of all tomentelloid EM fungi results from the incorporation of melanin, a natural dark pigment and common fungal wall component known to act as a protective interface between fungal metabolism and biotic and abiotic environmental stressors (Bell and Wheeler 1986; Butler and Day 1998; Kõljalg et al. 2000). An ecological role for melanin in EM fungi is still lacking; however, it is known that melanin in the cell wall of EM fungi can contribute to their heavy metal tolerance (Gadd 1993) and consequently enhance their competitiveness and establishment in polluted environments (Regvar et al. 2010). Bois et al. (2006) observed that excretion of yellowish phenolic-like compounds (e.g. extracellular melanin) by *Suillus tomentosus* increased with increasing NaCl concentrations present in the medium. Droplets of the same colour appeared on the surface of the mycelium. The authors suggest that the production and exudation of metabolites could be used for external osmotic adjustment to avoid the need of internal adjustment by the accumulation of Na and/or Cl (Bois et al. 2006). *Geopora* sp. were found on the roots of willow clones growing in unfavourable soil conditions; e.g. two *Salix viminalis* clones and a *S. viminalis* x *caprea* hybrid clone growing in fly ash (Hrynkiewicz et al. 2009a), and on *S. linearistipularis* roots in alkaline-saline soil (Ishida et al. 2009). This fungal taxa belongs to the subphylum of Pezizomycotina and the family Pyronemataceae. Pezizales species are often the dominant members of EM fungal communities in early succession ecosystems and following disturbances (Gehring et al. 1998). Thick-walled chlamydospores and ascospores may be reasons for their ability to persist under unfavourable environmental conditions (Tedersoo et al. 2006a). Pezizalean species have been discovered as EM symbionts in locally disturbed patches in forests with high pH and low organic matter (Petersen 1985; Dissing 2000), which might explain why the observed morphotype was able to survive in saline soil with a relatively high pH level (7.5–8.5) and formed an ectomycorrhizae with the willow clone in our experiment.

*Helotiales* sp., from the same subphylum as *Geopora* sp.—Pezizomycotina, was observed only in one season (autumn) on the roots of *B. pendula*. Helotiales is an order of Ascomycota, where many EM lineages await discovery (Tedersoo et al. 2009b). Because they are more commonly described as root endophytes and occasionally detected as ericoid mycorrhizal fungi, they most likely form other types of root-associated biotrophic relations with plants or represent secondary colonisers of EM root tips (Tedersoo et al. 2009b). Thus far, records of any sexual structures are lacking for seven out of eight helotialean lineages that strongly hamper their formal description, isolation in pure culture, and subsequent manipulative studies (Tedersoo et al. 2010). However, a yellowish EM morphotype with an ascomycete mantle anatomy, consistently identified as a member of Helotiales, was found on *Picea abies* (Tedersoo et al. 2008b), and eight species of Helotiales formed ectomycorrhizae with Australian hosts Tedersoo et al. (2008a, 2009a). These species were grouped into four well-supported lineages that are distantly related to the EM taxa in the Northern Hemisphere and to any root endophytes (Tedersoo et al. 2009b). Further investigations should help to clarify the unclear position of this taxa. The presence of this fungal strain in unfavourable saline conditions indicates their important role in plant-fungus interactions under abiotic stress.

The only representatives of the family Agaricales—*H. collariatum* Sc-A—was observed only once on the roots of *S. caprea. Hebeloma* spp. belong to the early-stage fungi (e.g. Mason et al. 1983) and are well-known colonisers of EM plants in disturbed or primary habitats (Jumpponen and Trappe 1998; Nara et al. 2003), which suggests that the EM fungus community is at an early successional stage with a relatively low number of fungal taxa. Relatively high colonisation with *Hebeloma* spp. was identified on *S. caprea* in heavy metal contaminated sites in Germany (Hrynkiewicz et al. 2008) and on *S. alba* in riparian edge forests in the Netherlands (Paradi and Baar 2006). Because EM root tips colonised by *H. collariatum* were identified only in the autumn in the present study (on the roots of *S. caprea*), we speculate that this genus can belong to periodic symbionts present in saline soils.

## Conclusions

The number of EM was different and depended on the tree species and season. As a major parameter, influencing the abundance of EM associated with the investigated tree species was salinity rather than level of P, K and other studied components. EM fungal density and diversity was low; however, the identified EM fungi, *Tomentella* sp., *Hebeloma* sp., *Geopora* sp. and *Helotiales* sp., can be classified to the group of species highly adapted to saline conditions.
